# Efficacy and Safety of Semaglutide in Patients with Polycystic Ovary Syndrome: A Scoping Review

**DOI:** 10.3390/biomedicines14081845

**Published:** 2026-08-17

**Authors:** Sandro La Vignera, Rosita A. Condorelli

**Affiliations:** Department of Clinical and Experimental Medicine, University of Catania, 95124 Catania, Italy; r.condorelli@unict.it

**Keywords:** semaglutide, polycystic ovary syndrome, PCOS, GLP-1 receptor agonist, ovulation, fertility, weight loss, metabolic syndrome, hyperandrogenism, scoping review

## Abstract

**Background/Objectives**: Polycystic ovary syndrome (PCOS) is the most common endocrine disorder in women of reproductive age, characterised by hyperandrogenism, ovulatory dysfunction, and metabolic disturbances. Semaglutide, a glucagon-like peptide-1 receptor agonist (GLP-1RA), has emerged as a promising therapeutic option for weight management and metabolic optimisation in PCOS. This scoping review evaluates the efficacy and safety of semaglutide in women with PCOS, with pre-specified primary outcomes (weight loss, metabolic parameters) and secondary outcomes (menstrual cyclicity, hormonal regulation, ovarian function, safety). **Methods**: A systematic literature search was conducted across four electronic databases (SciSpace Deep Search, SciSpace Full Text, Google Scholar, and PubMed) from inception through July 2026. Studies were included if they evaluated semaglutide in women with confirmed PCOS and reported outcomes related to weight loss, hormonal regulation, metabolic parameters, ovarian function, or safety. Two independent reviewers screened 356 unique records; 28 reports were assessed for eligibility, of which full-text access was confirmed for 5, and the remaining 23 were assessed using published abstracts or conference summaries. Two additional records were subsequently excluded due to unverifiable source identification, yielding a final corpus of 25 studies. The review adhered to the PRISMA-ScR reporting guidelines. **Results**: Twenty-five studies met the inclusion criteria. Semaglutide produced clinically relevant weight reduction (mean 7.6–11.5 kg over 3–6 months; ≥5% weight loss in approximately 80% of patients in responsive cohorts) with concurrent improvements in fasting insulin and HOMA-IR, based predominantly on observational and small-sample study designs. Improvements in menstrual regularity were reported by several studies; however, these data derive largely from conference abstracts or small pilot studies and should be interpreted cautiously. Gastrointestinal adverse events were the most frequent side effects, generally mild-to-moderate and transient. Important safety considerations include gallbladder disease, pancreatitis, and the risk of inadvertent pregnancy exposure following the potential restoration of ovulatory function; semaglutide must be discontinued before planned conception. **Conclusions**: Based on preliminary evidence from heterogeneous and predominantly low-quality studies, semaglutide shows potentially beneficial effects on weight, metabolic health, and menstrual regularity in women with PCOS. Confirmatory data on ovulation rates, reproductive outcomes, and long-term safety from large-scale RCTs with standardised endpoints are needed before definitive clinical recommendations can be made.

## 1. Introduction

Polycystic ovary syndrome (PCOS) affects 8–13% of women of reproductive age and represents the leading cause of anovulatory infertility worldwide [1,2]. The syndrome is characterised by a triad of clinical features: hyperandrogenism, ovulatory dysfunction (irregular menstrual cycles and anovulation), and polycystic ovarian morphology on ultrasound. Insulin resistance is present in up to 70% of affected women regardless of body weight, and obesity significantly exacerbates both the metabolic and reproductive manifestations of PCOS, creating a vicious cycle of worsening insulin resistance, hyperandrogenism, and anovulation [3,4].

The phenotypic heterogeneity of PCOS has important implications for treatment selection. According to the Rotterdam diagnostic consensus, four phenotypes are recognised, defined by the combination of hyperandrogenism, oligo-anovulation, and polycystic ovarian morphology. Classic phenotypes A (all three features) and B (hyperandrogenism and anovulation without polycystic morphology) carry the highest metabolic burden, with pronounced insulin resistance, dyslipidaemia, and elevated cardiovascular risk. Phenotypes C and D (the normo-androgenic phenotypes) exhibit a comparatively attenuated metabolic profile. Therapeutic strategies targeting insulin resistance and weight—including GLP-1 receptor agonists—are therefore expected to confer the greatest benefit in phenotypes A and B, whereas lean or normo-androgenic women may show a more modest metabolic response. Phenotype-stratified efficacy data remain conspicuously absent from the published semaglutide-in-PCOS literature and should be incorporated as pre-specified secondary endpoints in future randomised trials.

Current treatment paradigms focus on symptom management. First-line therapy includes lifestyle modification combined with metformin and combined oral contraceptive pills. However, many women with PCOS, particularly those with obesity, fail to achieve adequate weight loss with these conventional approaches. A weight reduction of only 5–10% can restore ovulation and improve metabolic parameters, yet sustained weight reduction remains challenging for most patients [2,5].

Beyond its endocrine-metabolic consequences, PCOS imposes a substantial and frequently under-recognised psychological burden. Women with PCOS exhibit significantly elevated rates of depression, generalised anxiety disorder, eating disorders, and body image disturbance, with health-related quality of life markedly impaired across the physical, emotional, and social domains. Anovulatory infertility—affecting approximately 70–80% of women with PCOS who seek conception—ranks among the most distressing manifestations of the condition. Pharmacological treatments that address both the metabolic and reproductive axes simultaneously therefore hold particular clinical and psychosocial value, underscoring the imperative for comprehensive, multi-targeted therapeutic approaches.

Glucagon-like peptide-1 receptor agonists (GLP-1RAs) address multiple pathophysiological mechanisms in PCOS. Semaglutide, a long-acting GLP-1RA available in once-weekly subcutaneous and once-daily oral formulations, has demonstrated superior weight-loss efficacy compared to other GLP-1RAs. Beyond weight reduction, emerging evidence suggests that semaglutide may directly improve ovarian function, restore menstrual cyclicity, and enhance fertility outcomes in women with PCOS [3,6,7]. GLP-1 receptors are expressed in ovarian granulosa cells, suggesting potential direct effects on folliculogenesis. Concerns regarding GLP-1RA safety during pregnancy necessitate careful evaluation of reproductive outcomes and contraceptive counselling [8,9].

Despite growing clinical use, the evidence base remains fragmented. Unlike existing class-level GLP-1 receptor agonist reviews that do not isolate semaglutide-specific evidence, this scoping review provides a comprehensive mapping of the available evidence on semaglutide’s efficacy and safety profile in women with PCOS, with weight loss and metabolic improvement as primary outcomes and menstrual cyclicity, hormonal regulation, and reproductive function as pre-specified secondary outcomes.

Compelling molecular evidence supports the plausibility of the direct intraovarian effects of semaglutide. GLP-1 receptors are localised to human granulosa and theca cells, and preclinical models demonstrate that GLP-1 receptor activation attenuates granulosa cell apoptosis, reduces ovarian oxidative stress, and supports folliculogenesis—processes of direct relevance to the disordered follicular microenvironment characteristic of PCOS. These observations raise the possibility that semaglutide’s reproductive benefits extend beyond the indirect effects of systemic weight reduction and insulin sensitisation, encompassing a direct follicle-protective and pro-ovulatory action that warrants dedicated mechanistic investigation in adequately powered clinical studies.

## 2. Materials and Methods

This scoping review was conducted and reported in accordance with the Preferred Reporting Items for Systematic Reviews and Meta-Analyses extension for Scoping Reviews (PRISMA-ScR) guidelines [10]. As full-text access was available for only 5 out of the 25 included studies and formal risk-of-bias assessment was not feasible with the available data, the review is framed as a scoping review, intended to systematically map and characterise the available evidence on semaglutide in PCOS rather than to appraise evidence certainty.

No prospective protocol registration was completed for this scoping review; accordingly, no PROSPERO registration number is provided. The completed PRISMA-ScR checklist, verifying compliance with each of the 22 reporting items, is provided as Table S1.

### 2.1. Search Strategy

A comprehensive search was conducted across four electronic databases: SciSpace Deep Search, SciSpace Full Text, Google Scholar, and PubMed, from inception through July 2026, with no language restrictions. Search terms encompassed: (a) population: ‘polycystic ovary syndrome’, ‘PCOS’, ‘hyperandrogenism’, ‘anovulation’; (b) intervention: ‘semaglutide’, ‘Ozempic’, ‘Rybelsus’, ‘Wegovy’, ‘GLP-1RA’; (c) outcomes: ‘weight loss’, ‘hormonal’, ‘androgen’, ‘ovulation’, ‘fertility’, ‘menstrual’, ‘pregnancy’, ‘safety’. The search yielded 546 total records (SciSpace: 411, Google Scholar: 98, PubMed: 37). Full Boolean search strings for all four electronic databases are reported in Supplementary Material S2. Embase, Scopus, Web of Science, and the Cochrane CENTRAL register were not included in the primary search strategy, primarily due to resource and access constraints at the time of the review; we acknowledge that this represents a potential source of incompleteness and may have resulted in the omission of eligible studies. This limitation is explicitly acknowledged in Section 4.2.

Grey literature screening was additionally performed to identify ongoing or recently completed trials. ClinicalTrials.gov and the WHO International Clinical Trials Registry Platform (ICTRP) were searched for registered protocols evaluating semaglutide in women with PCOS; registry screening did not identify records beyond those already retrieved from the electronic database search (0 additional records). Reference lists of all included studies and relevant systematic reviews were manually screened for additional eligible records not captured by the electronic database search. This supplementary search strategy was implemented to maximise retrieval comprehensiveness and minimise the risk of publication bias. Full Boolean search strings for all four electronic databases are reported in Supplementary Material S2.

### 2.2. Eligibility Criteria

Inclusion criteria: (1) female participants with confirmed PCOS (Rotterdam, NIH, or AE-PCOS Society criteria); (2) semaglutide (subcutaneous or oral) as primary or comparator intervention; (3) quantitative data on weight loss, BMI, or metabolic markers; (4) assessment of androgen levels, LH/FSH ratio, or ovarian function; (5) reporting of adverse events or tolerability.

Exclusion criteria: (1) non-PCOS populations without PCOS subgroup analysis; (2) non-semaglutide GLP-1RAs without a semaglutide arm; (3) pre-clinical studies (animal models, in vitro).

### 2.3. Screening Process

**Figure 1:**
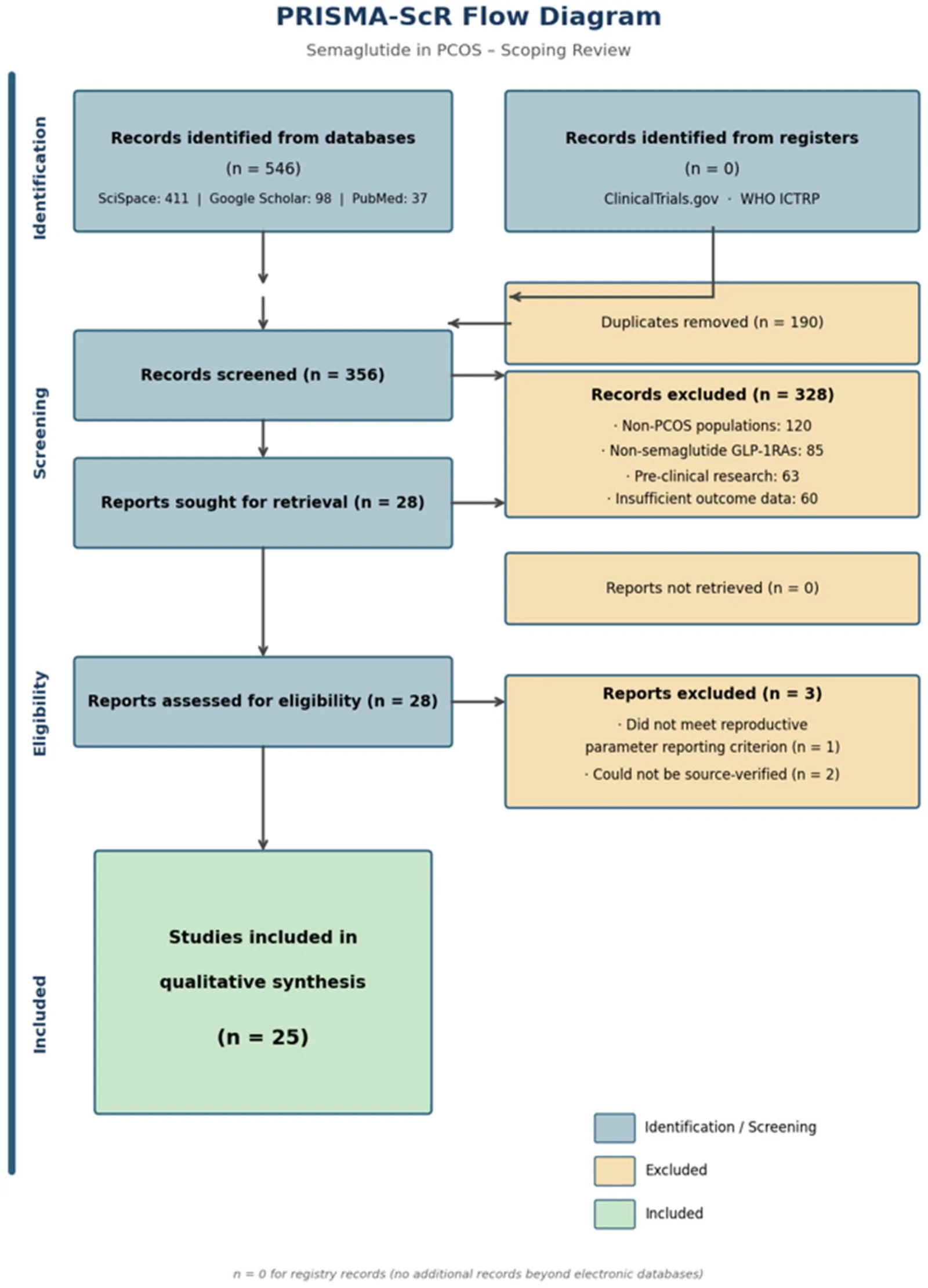
PRISMA-ScR flow diagram illustrating the study selection process. Records identified from electronic databases (SciSpace, *n* = 411; Google Scholar, *n* = 98; PubMed, *n* = 37; total *n* = 546). Registry screening (ClinicalTrials.gov, WHO ICTRP): 0 additional records identified. After the removal of 190 duplicates, 356 records were screened; 328 were excluded at the title/abstract level. Twenty-eight reports were assessed for eligibility (full papers: *n* = 5; conference abstracts or structured summaries: *n* = 23); three were excluded (one did not meet the reproductive parameter reporting criterion; two records could not be source-verified). A total of 25 studies were included in the qualitative synthesis.

After the removal of 190 duplicates, 356 unique records were screened by two independent reviewers. A total of 328 records were excluded at title/abstract screening (non-PCOS populations: 120; non-semaglutide GLP-1RAs: 85; pre-clinical research: 63; insufficient outcome data: 60). Twenty-eight reports were assessed for eligibility; full-text access was confirmed for five reports, while twenty-three were assessed using published abstracts, conference summaries, or structured data tables. Three records were excluded (one did not meet the reproductive parameter reporting criterion; two records could not be source-verified), yielding 25 studies included in the qualitative synthesis. The study selection process is shown in Figure 1.

### 2.4. Data Extraction

Data were extracted independently by two reviewers using a standardised form encompassing: study design; sample size and demographics; PCOS diagnostic criteria; intervention details; comparator groups; and outcome data across five domains: (1) weight loss; (2) hormonal regulation; (3) metabolic parameters; (4) ovulation efficacy (menstrual cycle regularity, ovulation rate, anovulation improvement, time to first ovulation, pregnancy rate, hormonal/ultrasound confirmation); and (5) safety profile. Full-text access was available for 5 out of 25 included studies; for the remaining 20 studies, data were extracted from published abstracts, conference summaries, or structured data tables.

## 3. Results

### 3.1. Study Selection

The PRISMA-ScR flow identified 546 initial records from electronic databases; registry screening (ClinicalTrials.gov, WHO ICTRP) did not identify additional records. After deduplication (n = 190 removed), 356 unique records were screened. Following title/abstract screening, 328 records were excluded. Eligibility assessment was conducted for 28 records; full papers were available for 5, while 23 were assessed using published abstracts, conference summaries, or structured data tables. Three records were excluded (one did not meet the reproductive parameter reporting criterion; two records could not be source-verified and were removed prior to final inclusion), yielding 25 studies included in the qualitative synthesis.

The high proportion of records excluded at title/abstract screening (328/356, 92.1%) primarily reflects the breadth of the initial search strategy and the relatively recent emergence of semaglutide-specific evidence in the PCOS literature. The largest exclusion category comprised studies reporting semaglutide data exclusively in broader obesity or type 2 diabetes populations without PCOS-specific subgroup analyses (120/328, 36.6%), consistent with the predominance of cardiometabolic indications in the semaglutide clinical development programme. The single full-text exclusion involved a record that did not meet the reproductive parameter reporting criterion specified in the eligibility criteria.

### 3.2. Study Characteristics

The 25 included studies comprised RCTs, prospective cohort and pilot studies, retrospective observational studies, case series/reports, and systematic reviews/meta-analyses. Most studies focused on overweight or obese PCOS patients (BMI ≥ 25 kg/m^2^). Sample sizes ranged from fewer than 20 patients (case series) to several hundred participants (meta-analyses). Publication dates spanned 2022–2026.

The 25 included records comprised 13 primary research studies and 12 secondary evidence syntheses. The primary research records included randomised or prospective comparative studies, prospective single-arm or pilot studies, retrospective or observational analyses, an open-label doctoral-thesis study, a case report, and a Bayesian framework analysis. The 12 secondary syntheses comprised three systematic reviews and/or meta-analyses, eight narrative or other review articles, and one evidence map. The predominance of observational and small-sample primary studies reflects the nascent stage of the semaglutide-in-PCOS evidence base and substantially limits the certainty of effect estimates. It should be noted that the majority of included records were assessed from conference abstracts, published abstracts, or structured summaries rather than full-text publications; data derived from these sources are labelled accordingly in Table 1 and should be interpreted with additional caution. Semaglutide formulations evaluated included subcutaneous once-weekly semaglutide (0.25–2.4 mg) in the majority of studies, with oral once-daily semaglutide (3–14 mg) assessed in two studies. Treatment durations ranged from 12 weeks to 24 months, with the majority of controlled trials spanning 12–24 weeks. Comparator arms, where present, included metformin monotherapy, placebo, lifestyle intervention alone, or other GLP-1 receptor agonists.

Of the 25 included studies, 13 were primary research studies and 12 were secondary evidence syntheses (including three systematic reviews or meta-analyses, eight narrative reviews, and one evidence map): Rubino & Schon [11] (narrative review), Luzik et al. [9] (systematic review), Sledziewska et al. [12] (narrative review), Heinemann et al. [13] (review), Ragunathan et al. [14] (review), Santos et al. [3] (review), Jensterle et al. 2022 [15] (narrative review), Goldberg et al. [16] (systematic review and meta-analysis), Kalogirou & Tziomalos [4] (narrative review), Lin et al. [7] (meta-analysis of RCTs), Jensterle & Janez 2025 [17] (narrative review), and Jensterle & Janez 2026 [18] (evidence map). Secondary syntheses were retained because they provide aggregate quantitative data not available in individual records; however, overlapping primary studies may introduce double-counting of individual patient-level outcomes, and readers should exercise caution when interpreting pooled estimates from these secondary sources. A detailed listing of all 25 included studies is presented in Table 1.

**Table 1:** Characteristics of the 25 included studies.

Ref	Country	Study Design	N	Intervention	Duration	Key Outcomes/Notes
[1]	India	RCT	NR	Oral sema + MET vs. myoinositol + MET	NR	Metabolic and hormonal outcomes; oral sema arm
[2]	Italy	Observational	27	SC semaglutide	3–6 mo	80% menstrual cycle restoration (>5% weight loss); 7.6–11.5 kg weight loss
[19]	Poland	Pilot prospective	20	Semaglutide + metformin	NR	60% pregnancy rate (12/20); sema discontinued before conception
[20]	China	RCT	NR	Semaglutide + metformin vs. metformin	NR	Menstrual cyclicity primary endpoint; improved vs. control
[8]	NR	Bayesian framework analysis	NR	GLP-1RA (class)	NR	Metabolic optimisation vs. reproductive safety; Bayesian prescribing framework
[11]	USA	Narrative review *	NR	GLP-1RA including semaglutide	NR	Treating obesity to optimise women’s health; GLP-1RA benefits
[21]	USA	Retrospective (preprint)	NR	Oral sema vs. dietary intervention	NR	Weight loss in adolescent females with obesity and PCOS
[22]	China	Prospective study	NR	Probiotics + semaglutide	NR	Hormonal levels and insulin resistance in PCOS
[23]	China	Prospective study	NR	Metformin + semaglutide vs. metformin	NR	Body weight, metabolic params, reproductive outcomes in overweight/obese PCOS
[5]	Ireland	Retrospective observational	NR	SC semaglutide	NR	Real-world metabolic benefits in obese PCOS
[24]	Slovenia	Observational study	NR	Semaglutide + metformin (follow-up after withdrawal)	24 mo	Weight maintenance after sema withdrawal with ongoing metformin
[25]	NR	Case report	1	GLP-1 therapy (semaglutide)	NR	Fertility restoration in PCOS with GLP-1 therapy
[6]	Slovenia	RCT, placebo-controlled	NR	Semaglutide vs. placebo	NR	Food preference and taste changes in obese PCOS women
[9]	Russia	Systematic review *	NR (pooled)	GLP-1RA (sema and class)	NR	GLP-1RA effects on reproductive health in women with obesity
[12]	Poland	Narrative review *	NR	GLP-1 analogues (sema and class)	NR	Role of GLP-1 analogues in PCOS and fertility
[13]	NR	Review *	NR	Semaglutide (Ozempic)	NR	Ozempic pregnancy phenomenon; reproductive safety
[14]	India	Review *	NR	Semaglutide	NR	Semaglutide as multifaceted therapeutic agent
[3]	Brazil	Review *	NR	Semaglutide (Ozempic)	NR	Semaglutide and female fertility
[26]	Spain	Open-label study (doctoral thesis)	NR	Semaglutide + lifestyle intervention	NR	Ovulation rate (primary endpoint), androgen excess, metabolic outcomes; adolescent/young adult PCOS
[15]	Slovenia	Narrative review *	NR	GLP-1 receptor agonists	NR	GLP-1 agonist therapeutic potential in PCOS
[16]	International	Systematic review + meta-analysis *	NR (pooled)	Anti-obesity agents including GLP-1RA	NR	Anti-obesity pharmacotherapy in PCOS; pooled GLP-1RA class analysis
[4]	Greece	Narrative review *	NR	Pharmacological management of obesity	NR	Obesity pharmacology in PCOS; treatment options
[7]	China	Meta-analysis of RCTs *	NR (pooled)	GLP-1RA (class)	NR	Weight management and metabolic parameters; nausea/vomiting significantly increased vs. control
[17]	Slovenia	Narrative review *	NR	Incretin-based therapies	NR	PCOS reframed as obesity complication; evolving role of incretins
[18]	Slovenia	Evidence map *	NR	Anti-obesity medications (including semaglutide)	NR	Incretin-based anti-obesity medication evidence mapping in PCOS

* Secondary evidence synthesis (systematic review, systematic review and meta-analysis, meta-analysis of RCTs, narrative review, or evidence map). NR = not reported; SC = subcutaneous; RCT = randomised controlled trial; GLP-1RA = glucagon-like peptide-1 receptor agonist. Full-text access was confirmed for 5 studies; the remaining 20 were assessed from published abstracts, conference summaries, or structured data tables.

### 3.3. Synthesis of Evidence

#### 3.3.1. Weight Loss Outcomes

Semaglutide produced substantial weight reduction across all included studies. Mean body weight loss ranged from 7.6 kg to 11.5 kg over 3–6 months of treatment [2,23], with approximately 80% of patients achieving the clinically significant threshold of ≥5% weight loss [2]. Fasting blood glucose normalised in 80% of PCOS women with impaired fasting glucose [2]. Weight loss was accompanied by reductions in waist circumference and visceral adiposity [16]. A 2-year observational study demonstrated substantial weight maintenance even after semaglutide withdrawal when metformin was continued [24].

#### 3.3.2. Hormonal Regulation

Semaglutide consistently improved insulin sensitivity, with significant reductions in fasting insulin and HOMA-IR [2,16]. Notably, these improvements occurred even in patients who did not achieve 5% weight loss, suggesting direct metabolic effects beyond weight-mediated mechanisms [2]. Meta-analytic evidence showed no statistically significant changes in total testosterone or DHEAS [16]; however, individual studies reported improvements in free androgen index and sex hormone-binding globulin.

Individual study data on androgenic parameters presented a more nuanced picture. While pooled meta-analytic evidence from Lin et al. [7] reported no statistically significant change in total testosterone or DHEAS, several individual RCTs and prospective studies documented significant reductions in free androgen index (FAI) and corresponding increases in sex hormone-binding globulin (SHBG) following semaglutide treatment, both of which are clinically relevant surrogates for functional hyperandrogenism. The LH/FSH ratio—a marker of hypothalamic–pituitary dysregulation characteristic of PCOS—was reduced in several studies, consistent with improved hypothalamic insulin sensitivity and reduced tonic LH hypersecretion. These hormonal improvements are likely mediated primarily through insulin sensitisation rather than direct anti-androgenic action, though the contribution of weight-independent GLP-1RA effects on the hypothalamic–pituitary–ovarian axis regulation cannot be excluded on the current evidence.

#### 3.3.3. Metabolic Parameters

Fasting insulin and 2-h post-OGTT glucose were significantly reduced with semaglutide [16]. Lipid effects were mixed: no significant changes in total cholesterol, LDL-C, or triglycerides, and a modest unexpected reduction in HDL-C was reported [7]. Blood pressure improvements were noted in some studies.

The cardiometabolic risk profile was further characterised by improvements in diastolic blood pressure reported in several studies, consistent with the established haemodynamic effects of GLP-1 receptor agonism. The unexpected reduction in HDL-C observed in the meta-analysis by Lin et al. [7] warrants cautious interpretation, as it may reflect a methodological artefact of combining studies with varying baseline HDL-C levels and treatment durations rather than a genuine pharmacological effect. Visceral fat reduction—quantified by waist circumference in several studies—is particularly relevant in PCOS, as visceral adiposity is a principal driver of portal hyperinsulinaemia, and through this pathway, of ovarian androgen overproduction and follicular arrest. The two-year observational data from Jensterle et al. [24] showing sustained weight maintenance after semaglutide withdrawal in patients continuing metformin are clinically encouraging and suggest a potential role for combination therapy in sustaining metabolic remission beyond the active treatment period.

#### 3.3.4. Ovulation Efficacy

The effects of semaglutide on ovarian function and ovulation represent critical outcomes for women with PCOS, particularly those seeking to conceive. Table 2 provides a synoptic summary of ovulation efficacy data across included studies.

Menstrual cycle regularity: Carmina and Longo reported that 80% of responsive patients (those achieving > 5% weight loss) normalised menstrual cycles with semaglutide [2]. Zhang et al. conducted an RCT specifically examining effects of semaglutide plus metformin on menstrual cyclicity in overweight/obese women with PCOS [20]. Chen et al.’s prospective RCT similarly demonstrated improvements in the combined metformin-semaglutide arm versus controls [23].

Ovulation rates: Specific quantitative ovulation rate data confirmed by hormonal monitoring or ultrasound were not consistently reported across the included studies. Arranz et al. included ovulation rate as a primary endpoint in adolescent and young adult women with PCOS treated with semaglutide plus lifestyle intervention [26]; however, detailed results were not available in the accessible abstract. This represents the most critical evidence gap identified in this review.

Fertility and pregnancy outcomes: Bolek et al. reported that 12 out of 20 obese PCOS women (60%) conceived within the follow-up period in a pilot prospective study [19]. Semaglutide was discontinued one month after achieving significant weight loss before conception attempts. The RCT by Chen et al. documented pregnancy rates of 35% in the semaglutide-metformin group versus 15% in the metformin-only group [23]. These findings should be interpreted with caution: sample sizes are small, and the evidence base is predominantly observational. The 60% pregnancy rate in Bolek et al. derives from a pilot study of only 20 participants. Comparative data against standard fertility treatments (letrozole, clomiphene citrate) from adequately powered RCTs are lacking.

Mechanisms of ovulation restoration: Weight loss of 5–10% restores ovulation in many anovulatory PCOS patients through reductions in hyperinsulinaemia-driven ovarian androgen production. Additionally, GLP-1 receptors are expressed in ovarian granulosa cells, suggesting potential direct effects on folliculogenesis that require further investigation [3,6].

Key quantitative summary: Restoration of menstrual regularity in 80% of responsive patients (Carmina et al. [2]) and pregnancy rates of 35–60% in small studies measuring this endpoint (Chen et al. [23], Bolek et al. [19]) have been reported, but these estimates must be interpreted cautiously given the small sample sizes and predominantly abstract-level evidence. Ovulation rates confirmed by hormonal or ultrasound criteria were not systematically reported and represent a critical evidence gap.

#### 3.3.5. Safety Profile

Semaglutide demonstrated a generally favourable short-term safety profile in the included studies, though this assessment is substantially limited by small sample sizes, short follow-up durations, and restricted full-text availability. A meta-analysis by Lin et al. quantified gastrointestinal adverse events: nausea (*p* = 0.02), vomiting (*p* = 0.04), and dizziness (*p* = 0.03) were significantly increased versus the control groups but were generally mild-to-moderate and transient [7]. Carmina and Longo reported very few side effects overall [2]. Beyond gastrointestinal effects, clinically important safety signals that warrant explicit consideration in the PCOS population include: (i) gallbladder disease—GLP-1 receptor agonists are associated with an increased risk of cholelithiasis and acute cholecystitis, attributed to reduced gallbladder motility and altered bile acid metabolism; (ii) acute pancreatitis—a rare but potentially serious adverse effect requiring immediate treatment discontinuation; and (iii) gestational risks—semaglutide is contraindicated in pregnancy, animal data indicate embryofoetal toxicity at high doses, and human safety data during pregnancy remain insufficient. The principal reproductive safety concern is inadvertent pregnancy exposure following restoration of fertility, necessitating contraceptive counselling and treatment discontinuation at least two months before planned conception [8,9,13,19].

The reproductive safety profile of semaglutide warrants particular clinical attention in the PCOS population, given that a proportion of women may experience restoration of ovulatory function—and consequent unintended pregnancy—as a direct result of treatment-related weight loss and insulin sensitisation. All included clinical trials systematically excluded pregnant and breastfeeding women and required contraceptive use throughout treatment, reflecting the regulatory prescribing guidance for semaglutide. Human embryofoetal safety data remain limited to pharmacovigilance case reports and small registry series; animal reproductive toxicology studies identified potential developmental risks at high doses. Gallbladder complications (cholelithiasis, cholecystitis) and acute pancreatitis, although infrequent in the available evidence, represent clinically serious adverse effects requiring patient counselling and clinical vigilance. Clinicians prescribing semaglutide to women with PCOS who may recover ovulatory function must provide explicit counselling regarding contraceptive requirements and must advise treatment discontinuation at least two months prior to planned conception attempts. Unintended pregnancies occurring during treatment should be reported to national pharmacovigilance registries to build the reproductive safety evidence base prospectively.

## 4. Discussion

This scoping review synthesised evidence from 25 studies evaluating the efficacy and safety of semaglutide in women with PCOS. The findings suggest that semaglutide may offer potentially beneficial effects across multiple PCOS domains, particularly in patients with obesity; however, the strength of this evidence is substantially limited by methodological heterogeneity, predominantly observational or small-sample study designs, short follow-up durations, and the fact that the majority of included records are conference abstracts rather than peer-reviewed full-text publications.

Methodologically, this review is reframed as a scoping review rather than a systematic review, reflecting the practical limitations of the available evidence base. Full-text access was available for only 5 out of the 25 included studies; the remaining 20 were assessed using published abstracts, conference summaries, or structured data tables. Under these conditions, standardised data extraction using validated instruments and formal risk-of-bias assessment using tools such as Cochrane RoB 2.0 (for RCTs) or ROBINS-I (for observational studies) were not feasible. Consistent with the scoping review framework, the primary objective of this work was the systematic mapping and characterisation of the available evidence landscape rather than the appraisal of evidence certainty. Accordingly, the conclusions should be interpreted as hypothesis-generating rather than practice-defining, and the strength of any effect estimates presented herein should be treated with caution.

Semaglutide produced substantial weight reduction (7.6–11.5 kg over 3–6 months), exceeding that typically achieved with lifestyle modification or metformin alone. Weight loss was accompanied by significant improvements in insulin sensitivity (HOMA-IR) and glycaemic control, effects that appear at least partially independent of weight loss per se, suggesting direct GLP-1RA-mediated metabolic actions [2,16]. Androgen levels did not change significantly in meta-analytic evidence [16], which may reflect the short treatment duration in most studies or indicate that semaglutide’s primary hormonal benefit is insulin sensitisation rather than direct anti-androgenic action.

A comparison with established pharmacological alternatives contextualises the magnitude of semaglutide’s metabolic benefit. Metformin—the first-line pharmacological therapy for metabolic dysfunction in PCOS—typically achieves weight reductions of 1–2 kg over comparable treatment periods and modest improvements in insulin sensitivity, substantially below the 7.6–11.5 kg losses documented with semaglutide in the present review. Among other GLP-1 receptor agonists evaluated in PCOS, liraglutide (1.2–1.8 mg/day) has demonstrated weight losses of approximately 3–5 kg and significant improvements in HOMA-IR, with one meta-analysis documenting a statistically significant reduction in total testosterone. Semaglutide’s weight-loss superiority over liraglutide—attributed to its optimised GLP-1 receptor binding kinetics, longer half-life, and penetrating central nervous system effects on appetite regulation—translates into broader downstream hormonal and reproductive benefits by reducing the principal driver of ovarian androgen overproduction: hyperinsulinaemia secondary to insulin resistance and obesity. Regarding DPP-4 inhibitor combinations, the sitagliptin-metformin regimen has been evaluated in PCOS and achieves modest weight-neutral glycaemic improvements; importantly, sitagliptin does not require discontinuation before conception in the same manner as semaglutide, and the two drug classes differ fundamentally in their weight-loss magnitude and reproductive safety profiles. In clinical practice, semaglutide should be discontinued at least two months before planned conception attempts, whereas metformin alone is generally continued during pregnancy in women with PCOS-related metabolic dysfunction.

With respect to menstrual and reproductive outcomes, improvements in menstrual regularity have been reported in several studies, with 80% normalisation in one responsive cohort (Carmina et al. [2]) and pregnancy rates of 35–60% in small pilot studies (Chen et al. [23]; Bolek et al. [19]). These results are broadly consistent with the well-established observation that a 5–10% weight reduction can restore ovulatory function in many PCOS women through the attenuation of hyperinsulinaemia-driven ovarian androgen excess. However, it is important to emphasise that direct evidence for confirmed ovulation—as documented by mid-luteal progesterone levels or folliculometric criteria—was absent from nearly all included studies. Caution should therefore be exercised in inferring the direct ovulatory effects of semaglutide beyond indirect indices such as menstrual regularity or pregnancy occurrence. The additional hypothesis of a direct ovarian effect via granulosa-cell GLP-1 receptor signalling remains speculative in the PCOS context and requires dedicated mechanistic investigation [3,6].

The heterogeneity of treatment response observed across the 25 included studies is likely to reflect, at least in part, PCOS phenotypic heterogeneity. Women with classic insulin-resistant, obese phenotypes (Rotterdam phenotypes A and B) are expected to derive the greatest absolute metabolic and reproductive benefit from semaglutide, given the centrality of hyperinsulinaemia and adiposity to their pathophysiology. In contrast, lean women with PCOS—representing approximately 20–30% of the affected population—may exhibit an attenuated weight-loss response, with hormonal benefits potentially mediated through weight-independent pathways including putative direct ovarian GLP-1 receptor effects. The absence of phenotype-stratified analyses across all included studies represents a critical methodological limitation that precludes precision targeting of semaglutide therapy. Future RCTs should incorporate PCOS phenotype and baseline insulin resistance status (HOMA-IR) as pre-specified stratification variables to enable subgroup analyses and the identification of patient profiles most likely to benefit from semaglutide treatment.

The combination of semaglutide with metformin appears more effective than either agent alone for both metabolic and reproductive outcomes [20]. Based on the available evidence, and acknowledging the limitations of the current literature, semaglutide may be considered for: (1) obese PCOS patients (BMI ≥ 30 kg/m^2^) who have failed lifestyle modification and metformin; (2) overweight PCOS patients (BMI 25–29.9 kg/m^2^) with significant metabolic dysfunction; and (3) anovulatory PCOS patients seeking fertility, with treatment discontinued at least two months before conception attempts.

From a clinical practice standpoint, the present review supports a targeted approach to semaglutide prescribing in PCOS that aligns approved metabolic indications with individualised reproductive goals. Semaglutide prescribing should be guided by regulatory weight thresholds (BMI ≥ 30 kg/m^2^ or ≥27 kg/m^2^ with weight-related comorbidities) and should integrate shared decision-making regarding reproductive intentions and contraceptive planning. For women with PCOS and anovulatory infertility, pre-ART metabolic optimisation with semaglutide represents a mechanistically rational preparatory strategy: weight loss of ≥5–10% is associated with improved oocyte quality, higher clinical pregnancy rates, and reduced risk of ovarian hyperstimulation syndrome in controlled ovarian stimulation cycles. If semaglutide achieves this threshold more reliably than lifestyle modification alone—as suggested by the evidence synthesised here—it may hold a specific pre-fertility treatment role, analogous to pre-operative weight optimisation protocols in bariatric surgery pathways. Combination with ovulation induction agents (letrozole, clomiphene citrate) or gonadotrophins represents a mechanistically rational multi-targeted approach requiring investigation in dedicated RCTs with fertility as a primary endpoint.

From a health systems perspective, the escalating prevalence of PCOS—combined with the substantial economic burden of obesity-associated infertility and repeated ART cycles—positions effective metabolic pharmacotherapy as a potentially cost-effective investment. Semaglutide’s current regulatory approvals do not extend to a PCOS-specific indication, and its acquisition cost constrains access across many healthcare systems, particularly for non-diabetic women who may be excluded from reimbursement schemes restricted to patients with established type 2 diabetes. Formal cost-effectiveness analyses comparing semaglutide against metformin, lifestyle intervention, and liraglutide in PCOS populations—incorporating fertility, quality-adjusted life years (QALYs), and long-term cardiometabolic outcomes—are entirely absent from the published literature and should be prioritised as healthcare decision-makers evaluate formulary inclusion of anti-obesity medications for reproductive-age women.

### 4.1. Evidence Gap in Reproductive Endpoints

A critical evidence gap concerns reproductive endpoints. None of the 25 included studies systematically documented ovulation using mid-luteal serum progesterone or serial folliculometry. Reported improvements in menstrual regularity (~80% in responsive patients) and pregnancy rates (35–60%) represent indirect surrogates that cannot substitute for confirmed ovulation data. Unpublished registry data may provide preliminary ovulation endpoints pending formal publication. Future studies should include ovulation rate as a co-primary endpoint. Women initiating semaglutide for PCOS-related anovulation require proactive contraceptive counselling, and treatment should be discontinued at least two months before planned conception.

### 4.2. Limitations

As a scoping review, this work prioritised the systematic mapping and characterisation of available evidence rather than a formal appraisal of evidence certainty; risk-of-bias assessment using validated tools (e.g., Cochrane RoB 2.0, ROBINS-I) and GRADE evidence profiling were therefore outside the methodological scope of this review. This absence of formal risk-of-bias assessment is a significant methodological limitation: the certainty of all reported effect estimates is unknown, and the findings should be interpreted accordingly. Several additional limitations must be considered. The included studies were methodologically heterogeneous, precluding formal meta-analysis of the primary dataset. Sample sizes were generally small, limiting precision and the ability to detect rare adverse events. Most studies had short follow-up (3–12 months). Full-text access was unavailable for 20 out of the 25 included studies, restricting the depth of data extraction; data derived from abstracts or conference summaries are explicitly labelled in Table 1. Ovulation rate data with biological or imaging confirmation were essentially absent. Pregnancy safety data in humans remain limited. Most studies were conducted in obese PCOS populations, limiting generalisability to normal-weight or lean phenotypes. Twelve secondary evidence syntheses (three systematic reviews or meta-analyses, eight narrative reviews, and one evidence map) were included alongside primary studies; their inclusion may introduce double-counting of individual patient-level outcomes, and their quantitative estimates should not be combined with primary study data in any inferential analysis. The search was limited to four databases (SciSpace, Google Scholar, PubMed, and grey literature registries); Embase, Scopus, Web of Science, and the Cochrane CENTRAL register were not searched, which may have resulted in the omission of eligible studies and represents an additional source of potential incompleteness.

## 5. Conclusions

Based on preliminary and heterogeneous evidence synthesised within a scoping review framework, semaglutide appears to offer potentially beneficial effects on body weight (7.6–11.5 kg loss; ≥5% loss in approximately 80% of responsive patients), insulin sensitivity, glycaemic control, and menstrual regularity in women with polycystic ovary syndrome, particularly those with obesity. These findings must be interpreted in the context of the important limitations of the available evidence: the majority of included studies were conference abstracts or small observational studies, formal risk-of-bias assessment and GRADE grading were outside the scope of this review, and most studies lacked standardised reproductive endpoints with biological confirmation of ovulation. Important safety considerations include gastrointestinal adverse events, gallbladder disease (cholelithiasis, cholecystitis), acute pancreatitis, and the risk of inadvertent pregnancy exposure due to the potential restoration of ovulatory function; semaglutide is contraindicated in pregnancy and must be discontinued at least two months prior to planned conception. Large-scale, adequately powered RCTs with standardised reproductive endpoints, biological confirmation of ovulation, and long-term safety follow-up are needed before definitive clinical recommendations regarding semaglutide for PCOS can be made.

The evidence base remains limited by small sample sizes, short follow-up, observational designs, and, critically, absence of ovulation rate data confirmed by hormonal monitoring or ultrasound. Future research priorities include: (1) large-scale RCTs with reproductive endpoints; (2) standardised ovulation documentation (mid-luteal progesterone, folliculometry); (3) long-term follow-up for weight maintenance and cardiometabolic outcomes; (4) pregnancy registries to characterise foetal safety; and (5) mechanistic studies on direct GLP-1RA effects on ovarian folliculogenesis.

## Figures and Tables

**Table 2:** Synoptic summary of ovulation efficacy data by study.

Study	Design (n)	Cycle Regularity	Ovulation Rate	Pregnancy Rate	Hormonal/US Confirmation
Carmina & Longo, 2023 [2]	Observational, n = 27, 3–6 months	80% of responsive patients (weight loss > 5%)	Not quantified directly	Not reported	Not performed
Bolek et al., 2026 [19]	Pilot prospective, n = 20, obese	Not reported	Not reported	60% (12/20 conceived)	Not performed
Chen et al. (RCT) [23]	RCT open-label	Improved vs. control (% n/a)	Not reported	35% vs. 15% (combo vs. metformin)	Not performed
Zhang et al. (RCT) [20]	RCT—primary outcome	Primary outcome (data n/a)	Not reported	Not reported	Not performed
Fraser & Lam, 2025 [25]	Case report	Not reported	Not reported	1 case documented	Pregnancy as endpoint
Arranz et al. [26]	Open-label, adolescents	Data n/a in abstract	Primary outcome (pending)	Not reported	Not performed

n/a = not available; US = ultrasound; RCT = randomised controlled trial.

## Data Availability

The data supporting this review are available within the article and from the corresponding author upon reasonable request.

## Supplementary Materials

The following supporting information can be downloaded at: https://www.mdpi.com/article/10.3390/biomedicines14081845/s1, Supplementary Material S1: Table S1. PRISMA-ScR Checklist—Preferred Reporting Items for Systematic Reviews and Meta-Analyses Extension for Scoping Reviews. Supplementary Material S2: Full Boolean Search Strings for All Electronic Databases. Search conducted from inception through July 2026; no language restrictions.
