# Supplementary material for: Efficacy and Safety of Semaglutide in Patients with Polycystic Ovary Syndrome: A Scoping Review
Source: Biomedicines. 2026 Aug 17;14(8):1845. doi: 10.3390/biomedicines14081845 (PMC13510204; doi:10.3390/biomedicines14081845)
Supplement: Supplementary file 1 [file biomedicines-14-01845-s001.zip › biomedicines-4449422-supplementary.pdf]

Supplementary Material S1

Table S1. *PRISMA-ScR Checklist — Preferred Reporting Items for Systematic Reviews and Meta-Analyses Extension for Scoping Reviews.*

Section/Topic	Item #	Checklist Item	Reported	Location in Manuscript
Title	1	Identify the report as a scoping review.	Yes	Title page
Abstract	2	Provide a structured summary that includes (as applicable): background; objectives; eligibility criteria; sources of evidence; charting methods; results; and conclusions that relate to the review questions and objectives.	Yes	Abstract
Introduction: Rationale	3	Describe the rationale for the review in the context of what is already known. Explain why the review questions/objectives lend themselves to a scoping review approach.	Yes	Introduction
Introduction: Objectives	4	Provide an explicit statement of the objective(s) and question(s) to be addressed with reference to their key elements (e.g. population, concept, and context) or other relevant key elements.	Yes	Introduction
Methods: Protocol and registration	5	Indicate whether a review protocol exists; state if and where it can be accessed (e.g. a web address); and if available, provide registration information including the registration number.	Yes	Methods, Protocol and Registration
Methods: Eligibility criteria	6	Specify characteristics of the sources of evidence used as eligibility criteria (e.g. years considered, language, and publication status), and provide a rationale for the decisions.	Yes	Methods, Eligibility Criteria
Methods: Information sources	7	Describe all information sources in the search (e.g. databases with dates of coverage and contact with study authors to identify additional sources) and the date the most recent search was executed.	Yes	Methods, Information Sources
Methods: Search	8	Present the full electronic search strategy for at least one database, including any limits used, such that it could be repeated.	Yes	Supplementary Material S2
Methods: Selection of sources of evidence	9	State the process for selecting sources of evidence (i.e. screening and eligibility) included in the scoping review.	Yes	Methods, Selection of Sources of Evidence
Methods: Data charting process	10	Describe the methods of extracting data from the included sources of evidence (e.g. whether performed independently or in duplicate and any processes for obtaining and confirming data from investigators).	Yes	Methods, Data Charting Process
Methods: Data items	11	List and define all variables for which data were sought and any assumptions and simplifications made.	Yes	Methods, Data Items
Methods: Critical appraisal of individual sources of evidence	12	If done, provide a rationale for conducting a critical appraisal of included sources of evidence; describe the methods used and how this information was used in any potential synthesis.	NA	Not applicable (consistent with scoping review methodology)

Section/Topic	Item #	Checklist Item	Reported	Location in Manuscript
Methods: Synthesis of results	13	Describe the methods of handling and summarising the data that were charted.	Yes	Methods, Synthesis of Results
Results: Selection of sources of evidence	14	Give numbers of sources of evidence screened, assessed for eligibility, and included in the review, with reasons for exclusions at each stage, ideally using a flow diagram.	Yes	Results; Figure 1 (PRISMA-ScR Flow Diagram)
Results: Characteristics of sources of evidence	15	For each source of evidence, present characteristics for which data were charted and provide the citations.	Yes	Results; Table 1
Results: Critical appraisal within sources of evidence	16	If done, present data on critical appraisal of included sources of evidence (see item 12).	NA	Not applicable
Results: Results of individual sources of evidence	17	For each included source of evidence, present the relevant data that were charted that relate to the review objectives.	Yes	Results; Table 1
Results: Synthesis of results	18	Summarise and/or present the charting results as they relate to the review questions and objectives.	Yes	Results (narrative synthesis)
Discussion: Summary of evidence	19	Summarise the main results (including an overview of concepts, themes, and types of evidence available), link to the review questions and objectives, and consider the relevance to key groups.	Yes	Discussion
Discussion: Limitations	20	Discuss the limitations of the scoping review process.	Yes	Limitations
Discussion: Conclusions	21	Provide a general interpretation of the results with respect to the review questions and objectives, as well as potential implications and/or next steps for future scoping reviews, systematic reviews, or other types of knowledge syntheses.	Yes	Conclusions
Funding	22	Describe sources of funding for the included sources of evidence, as well as sources of funding for the scoping review. Describe the role of the funders of the scoping review.	Yes	Funding

Supplementary Material S2

Full Boolean Search Strings for All Electronic Databases. Search conducted from inception through July 2026; no language restrictions.

SciSpace Deep Search

("polycystic ovary syndrome" OR "PCOS" OR "hyperandrogenism" OR "anovulation") AND ("semaglutide" OR "Ozempic" OR "Rybelsus" OR "Wegovy" OR "GLP-1RA") AND ("weight loss" OR "hormonal" OR "androgen" OR "ovulation" OR "fertility" OR "menstrual" OR "pregnancy" OR "safety")

Date range: inception to July 2026; no language restrictions.

Records retrieved: 411

SciSpace Full Text Search

("polycystic ovary syndrome" OR "PCOS" OR "hyperandrogenism" OR "anovulation") AND ("semaglutide" OR "Ozempic" OR "Rybelsus" OR "Wegovy" OR "GLP-1RA") AND ("weight loss" OR "hormonal" OR "androgen" OR "ovulation" OR "fertility" OR "menstrual" OR "pregnancy" OR "safety")

Date range: inception to July 2026; no language restrictions.

Records retrieved: included within SciSpace totals

Google Scholar

("polycystic ovary syndrome" OR PCOS OR hyperandrogenism OR anovulation) AND (semaglutide OR Ozempic OR Rybelsus OR Wegovy OR "GLP-1RA") AND ("weight loss" OR hormonal OR androgen OR ovulation OR fertility OR menstrual OR pregnancy OR safety)

Date range: inception to July 2026; no language restrictions.

Records retrieved: 98

PubMed

("polycystic ovary syndrome"[MeSH Terms] OR "PCOS"[Title/Abstract] OR "hyperandrogenism"[Title/Abstract] OR "anovulation"[Title/Abstract]) AND ("semaglutide"[All Fields] OR "Ozempic"[All Fields] OR "Rybelsus"[All Fields] OR "Wegovy"[All Fields] OR "GLP-1 receptor agonist"[All Fields] OR "GLP-1RA"[Title/Abstract]) AND ("weight loss"[Title/Abstract] OR "androgen"[Title/Abstract] OR "ovulation"[Title/Abstract] OR "fertility"[Title/Abstract] OR "menstrual"[Title/Abstract] OR "pregnancy"[Title/Abstract] OR "safety"[Title/Abstract])

Date range: inception to July 2026; no language restrictions.

Records retrieved: 37
